# Complete mitochondrial genome of biraphid benthic diatom, *Navicula ramosissima* (Naviculales, Bacillariophyceae)

**DOI:** 10.1080/23802359.2016.1198997

**Published:** 2016-07-23

**Authors:** Sung Min An, Jae Hoon Noh, Hyee Ryun Lee, Dong Han Choi, Jung Ho Lee, Eun Chan Yang

**Affiliations:** a Marine Ecosystem and Biological Research Center, Korea Institute of Ocean Science & Technology, Ansan, Korea;; b Department of Biology Education, Daegu University, Gyeongsan, Korea

**Keywords:** Diatoms, mitochondrial genome, Naviculales, *Navicula romosissima* TA439

## Abstract

The complete mitochondrial DNA of biraphid benthic diatom, *Navicula ramosissima* TA439 was sequenced and characterized. The circular mitogenome contains 67 genes in 48,652 bp (31.1% GC), including 41 protein-coding, 24 transfer RNA (tRNA) and 2 rRNA genes. Twenty-four protein-coding sequences (CDS, 59%) have start with ATG codon and 17 CDS start with alternatives such as ATA (5), ATT (6), TTA (5) and TTG (1). The GC content of tRNA genes (42.1%) is relatively higher than those of the rRNA (35.2%) and CDS (30.5%). Three genes are consisted of multiple exons and introns, i.e. *cox1* (three exons, two introns), *rps11* (two exons, one intron), *rrl* (four exons, three introns). Phylogeny of diatoms based on mitogenome data (34 CDS, 8530 amino acids combined) supports the monophyly of Naviculales, including *N. ramosissima* (Naviculaceae), *Berkeleya fennica* (Berkeleyaceae), *Fistulifera solaris* (Stauroneidaceae) and *Phaeodactylum tricornutum* (Phaeodactylaceae). Mitogenome data may be useful for phylogenetic study of the diatoms and stramenopiles.

*Navicula ramosissima* (C.Agardh) Cleve is ubiquitous species in brackish and marine water, and is dominant during the winter months (Werner 1977; Snoeijs & Kautsky 1989). The species is included in biraphid motile diatom because individual cell moves and grows colonially in branched mucilaginous tube (Lobban & Navarro 2013). The species is one of the most numerous diatoms in tidal mudflat of the west coast of Korea, thus subject to primary production monitoring researches. However, no mitochondrial data available yet, only nuclear rRNAs (18S and 28S) and plastid genes (*rbcL* and *16S rRNA*) were reported to public. In the present study, we determined novel mitogenome of *N. ramosissima* TA439 isolated from the Geunsoman, Taean (36°44′12.06″ N 126°10′47.52″ E), Korea (strain available upon request). We followed next-generation sequencing strategy as described in An et al. (2016) and Yang et al. (2015).

The complete mitogenome (mtDNA) of *N. ramosissima* TA439 (GenBank accession no. KX343079) indicates a 48,652 bp of circular genome with 31.1% GC content. Total 67 genes are encoded in 40,335 bp (83% of total size) exclude introns, including of 41 protein-coding sequences (CDS; i.e. 18 respiratory, 16 ribosomal and 7 hypothetical protein-coding genes; total 34,236 bp with 31.5% GC), 2 ribosomal RNA (*rrl* and *rrs*; total 4286 bp exclude introns with 35.2% GC) genes for large and small subunits, and 24 transfer RNA (tRNA; total 1813 bp with 42.1% GC) genes. The ATG is the most common start codon used in 24 protein-coding sequences (CDS; 59% of total CDS) and followed by ATT (six CDS), ATA (five CDS), TTA (five CDS), and TTG (one CDS) codons. All CDS use the stop codon either TAA (31 CDS; 76%) or TAG (10 CDS). Three genes include multiple exons and introns, i.e. *cox1* (three exons and two introns), *rps11* (two exons, one intron), *rrl* (four exons, three introns). Two *cox1* introns (2439 bp, 2325 bp) harbour intronic ORF such as ORF717 and ORF750. The intron-2 (2422 bp) and intron-3 (2355 bp) of *rrl* introns harbour ORF558 and ORF218, respectively. No intronic ORF encoded in *rrl* intron-1 (834 bp) and *rps11* intron (37 bp). All intronic ORF matched with diatoms’ intronic ORF which encoded the Group II intron-specific reverse transcriptase/muturase, e.g. *orf79* of *Pseudo-nitzschia multiseries* (GI: 836614283). The 24 tRNA genes, ranging from 69 to 89 bp in length, show typical cloverleaf secondary structures.

Translated amino acid sequences of *N. ramosissima* TA439 mitogenome used for phylogenetic analyses with those of all available diatoms, i.e. the best tree construction and bootstrap analysis under the maximum-likelihood (ML) criterion. The best phylogeny of 34 CDS concatenated data (8530 amino acids) supports the monophyly of Bacillariophyta (100% ML bootstrap support, MLB; Figure 1) and congruent with previous study (An et al. 2015). Naviculales is grouped with Bacillariales within Bacillariophycidae (100% MLB). Monophyletic Naviculales (100% MLB) included *N. ramosissima* (Naviculaceae), *B. fennica* (Berkeleyaceae), *F. solaris* (Stauroneidaceae), and *P. tricornutum* (Phaeodactylaceae). New phylogeny suggested that additional mitogenome data from representative lineages may be useful for the systematic study of diatoms.

**Figure 1.**
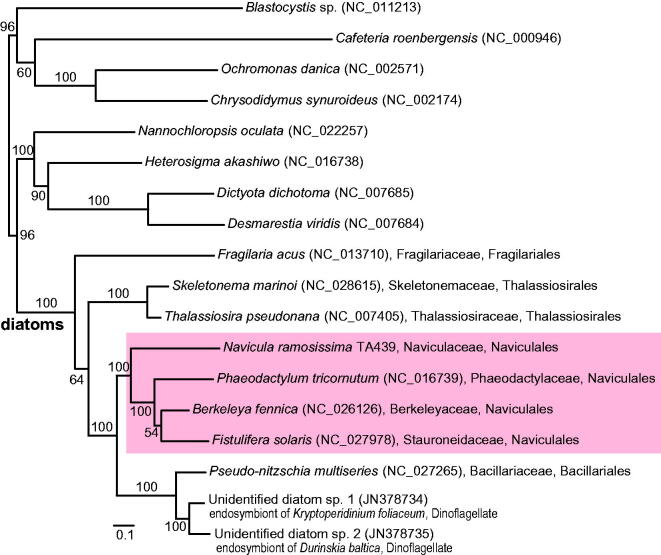
ML tree of the diatoms based on the mitochondrial genome data, 34 CDS concatenated data (8530 amino acids). The support value of each node calculated from ML bootstrap analysis, 100 non-parametric bootstrapping. Diatom species name were followed by the GenBank accession number in parenthesis and family and order names.
